# Characteristics of soil organic carbon fractions in four vegetation communities of an inland salt marsh

**DOI:** 10.1186/s13021-024-00248-2

**Published:** 2024-01-28

**Authors:** Manping Kang, ChengZhang Zhao, Min Ma, Xiaoya Li

**Affiliations:** 1 College of Geography and Environmental Science, Northwest Normal University, Lanzhou, 730070 China; 2 College of Geography and Environmental Science, Research Center of Wetland Resources Protection and Industrial Development Engineering of Gansu Province, Northwest Normal University, Lanzhou, 730070 China

**Keywords:** Salt marsh, Plant community type, Soil organic carbon, Reactive organic carbon, Physical and chemical properties of soil

## Abstract

**Background:**

The study of soil organic carbon characteristics and its relationship with soil environment and vegetation types is of great significance to the evaluation of soil carbon sink provided by inland salt marshes. This paper reports the characteristics of soil organic carbon fractions in 0–50 cm soil layers at four vegetation communities of the Qinwangchuan salt marsh.

**Results:**

(1) The soil organic carbon content of Phragmites australis community (9.60 ± 0.32 *g*/kg) was found to be higher than that of Salicornia europae (7.75 ± 0.18 *g*/kg) and Tamarix ramosissima (4.96 ± 0.18 *g*/kg) and Suaeda corniculata community (4.55 ± 0.11 *g*/kg). (2) The soil dissolved organic carbon, particulate organic carbon and soil microbial biomass carbon in 0–50 cm soil layer of Phragmites australis community were higher, which were 0.46 ± 0.01 *g*/kg, 2.81 ± 0.06 *g*/kg and 0.31 ± 0.01 *g*/kg, respectively. (3) Soil organic carbon was positively correlated with dissolved organic carbon, particulate organic carbon, and microbial biomass carbon, and negatively correlated with easily oxidized organic carbon. (4) Above-ground biomass has a strong direct positive effect on soil organic carbon, total nitrogen and pH have a strong direct positive effect on microbial biomass carbon content, pH and average density have a strong direct negative effect on easily oxidized organic carbon, and particulate organic carbon.

**Conclusions:**

The interaction between plant community characteristics and soil factors is an important driving factor for soil organic carbon accumulation in inland salt marshes.

## Background

Wetland is an important part of the terrestrial ecosystem carbon cycle, with high carbon storage capacity. Comprising one-third of the global organic soil carbon pool, wetlands are considered to represent one of the largest biological carbon pools and decisively affect global carbon cycling [1]. The inland salt marsh is a marsh wetland with over-wet or seasonal water, salinized soil, and halophytes in continental arid and semi-arid climates [2]. The inland salt marsh is a type of wetland ecosystem that exhibits specific ecological and hydrological processes and a highly heterogeneous environment [3]. Salt marsh has a special water-saturated anaerobic environment, which is characterized by high species diversity, high local productivity, high carbon burial rate, and low carbon decomposition rate [4]. Carbon sequestration in the salt marsh is an important part of the wetland carbon sink. The carbon capacity of salt marsh is stronger than that of other vegetation ecosystems in arid areas, and it is very sensitive to regional and global climate change [5]. To study the influencing mechanism of carbon sequestration in the vegetation-soil system of inland salt marsh wetlands, and to provide a scientific basis for revealing the mechanism of soil organic carbon accumulation in wetlands under the background of global change.

Soil is the main carbon pool in wetlands, accounting for 95% of carbon storage [6]. Soil organic carbon (SOC) is not only an important component of wetland soil but also plays an important role in regulating wetland carbon storage, primary productivity of the wetland ecosystem, and global climate change [7]. SOC is not only an important component of wetland soils but also plays an important role in regulating carbon storage, wetland ecosystem primary productivity, and global climate change [8]. Marsh hydrology is the main driving force that maintains the development and decline of wetlands [9]. Soil active organic carbon refers to the part of soil organic carbon that transfers quickly in soil, has unstable chemical properties, is easily oxidized, decomposed, and mineralized, and its morphology and spatial location have a high impact on the activities of plants and microorganisms [10]. Soil active organic carbon refers to the unstable part of the soil that is fast-moving, has poor stability, easy to oxidation, is easy to mineralization, and is highly active to plants and soil microorganisms [11]. According to different methods of separation and determination, soil active organic carbon can be characterized in various forms: dissolved organic carbon (DOC), easily oxidized organic carbon (EOC), microbial biomass carbon (MBC), particulate organic carbon (POC) [12]. EOC is the first part of organic carbon to be oxidized, is the fastest turnover of soil organic carbon component, and is also a sensitive index of soil organic matter dynamic change [13]. DOC is an organic carbon source that can be directly used by soil microorganisms. It has certain solubility, moves quickly in soil, and is easy to decompose and mineralize into carbon dioxide, which is released into the atmosphere or lost in water [14]. POC refers to the fraction of soil organic carbon that is bound to sand particles (53–2000 μm in diameter), and may be further bound to soil macro-aggregates and micro-aggregates, which is susceptible to the distribution of soil particles and roots [15]. Although the proportion of soil organic carbon active components in SOC is small, the changes in these components are more sensitive than SOC and can largely characterize the changes in soil SOC content. Therefore, they are of great significance for soil carbon pool balance, soil biochemistry, and soil fertility maintenance.

Due to long-term flooding or water supersaturation, wetlands accumulate more active organic carbon and are more sensitive to climate change [16]. In recent years, domestic scholars have studied the distribution and accumulation of soil organic carbon in wetlands mainly in coastal wetlands [17], Sanjiang Plain [18], Poyang Lake [19], and Zoigai Plateau [20, 21] and different wetland types in different regions. These studies showed that at the regional scale, the size and components of the soil carbon pool varied significantly among different wetland types under the influence of hydrothermal conditions. Even in the same area, due to the differences in wetland flooding frequency and plant community type, the distribution of soil organic carbon and active carbon components is different [22]. Foreign scholars have found that the effects of different plant community types on soil organic carbon and its active carbon components are complex [23]. On the one hand, the characteristics of the plant community, litter and exudates affect the quality and quantity of soil organic carbon input, and then affect the accumulation of soil organic carbon [24]. On the other hand, differences in soil physical and chemical properties, such as soil moisture content, pH, bulk density, and total nitrogen, can also affect microbial activity, plant growth, community height, density, and biomass allocation patterns, leading to changes in soil organic carbon production [25]. An inland salt marsh wetland is a wetland ecosystem with specific ecological and hydrological processes and a highly heterogeneous environment [26]. Soil organic carbon and its active carbon components have a profound impact on the ecosystem services of salt marshes by affecting the physical, chemical, and biological characteristics of soil [27]. There are few reports on soil organic carbon and its active carbon components under halophyte communities in inland salt marsh ecosystems, especially the processes and mechanisms affecting soil organic carbon and its active carbon components in salt marsh wetlands are not clear. Therefore, the study of soil organic carbon composition characteristics and influencing factors of different plant communities in inland salt marsh wetlands can clarify the impact of vegetation community types on wetland carbon sink function, and provide the scientific basis for the protection and restoration of inland salt marsh wetlands.

Qinwangchuan salt marsh wetland is a typical salt marsh wetland in Lanzhou, which has a remarkable ecological function, special protection value and high scientific research value. The study area is rich in plant species, mainly salt-tolerant and salt-secreting plants. Among them, *Phragmites australis* (*PA*), *Tamarix ramosissima* (*TR*), *Salicornia europaea* (*SE*), and *Suaeda corniculata* (*SC*) are the typical vegetation communities in the salt marsh. Each plant community is dominated by a certain species, with few associated species, forming a single vegetation-type wetland with a simple structure. The transformation and distribution of soil organic carbon and active carbon components in wetland ecosystems are closely related to hydrological processes and vegetation types. At present, many scholars have studied the ecosystem service trade-offs [28], Spatiotemporal variations and driving factors of habitat quality in the Qinwangchuan wetland [29]. It is urgent to reveal the characteristics and influencing mechanism of soil organic carbon and active carbon components of different plant communities in inland salt marsh wetlands. Based on this, the Qinwangchuan salt marsh wetland is taken as the research area, the selection of *PA*, *SE*, *TR,* and *SC* typical plant communities as the research object, analysis of different plant communities 0–50 cm soil organic carbon and carbon content of the active component. Pearson correlation and path analysis to explore the rational soil physical and chemical effects on soil organic carbon and active carbon components. Hypotheses were as follows. (1) Soil organic carbon and active carbon components were different in different plant communities. (2) Soil organic carbon difference and active carbon components were affected by vegetation community and soil factors. (3) There are direct and indirect effects of factors on the change of soil organic carbon and active carbon components. It is helpful to reveal the stability of the soil carbon pool in salt marsh and its influencing mechanisms and provide data support for maintaining the function of the inland salt marsh ecosystem.

## Methods

### Study sites and sampling

**Fig. 1:**
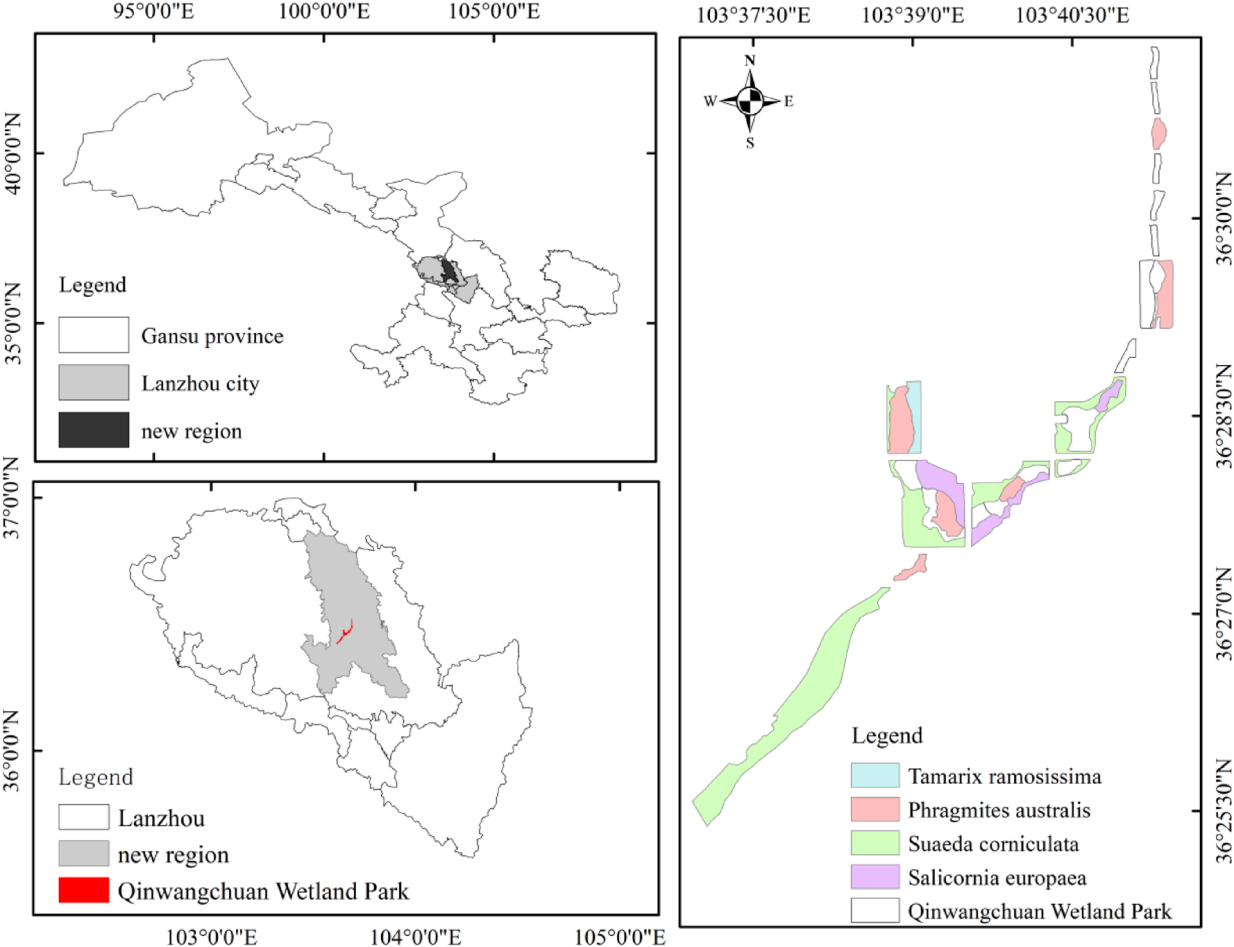
Locations of measured plots and the study area

Qinwangchuan Basin is located in the transition zone between the desert grassland area in the north and the typical grassland vegetation area in the middle of the Loess Plateau. It is the largest alluvial plateau basin in Lanzhou (Fig. 1). The overall topography is high in the north and low in the south, and the altitude is between 1800 and 2300 m. Qinwangchuan salt marsh wetland is an overflow area of natural precipitation in the Qinwangchuan Basin, irrigation water is diverted into the Qin Project and groundwater is underflow. After long-term climatic and hydrological processes and water environment effects, rare inland salt marshes in the Loess Plateau of the Longzhong region have been gradually developed, which is representative and typical of the arid and semi-arid salt marshes ecosystem in northwest China. Qinwangchuan National Wetland Park is 103°36ʹ28″—103°40ʹ13″E, 36°24ʹ52″-36°28ʹ44″N, located in the transitional zone between arid desert and Tengger Desert. The average annual temperature of the region is 6.9 ℃, the annual sunshine time is 2700 h, the average frost-free period is 126 days, the average annual rainfall is 265 mm, and the average annual evaporation is 1879 mm. It has a continental arid climate. The main plants in the Qinwangchuan salt marsh wetland are *Suaeda corniculata, Salicornia europaea, Phragmites australis, Tamarix ramosissima, Salsola collina, Atriplex patens, Tripolium vulgare and Sonchus oleraceus.*

### Experimental method and design

Based on field investigation, the relatively flat surface area in Qinwangchuan salt marsh wetland was selected as the sample site in September 2021. According to the different dominant species of plant communities in the plots, three typical halophyte communities (*PA, SE, and SC*) with similar topography, elevation, slope and aspect were selected, and three 10 m × 10 m plots were set for each community. Five 1 m × 1 m quadrats were set in each plot of *PA*, *SC*, and *SE*. five 2 m × 2 m quadrats were set in each plot of *TR*. A vegetation survey was conducted on four typical halophyte communities, including plant height, coverage and density, and aboveground biomass was collected.

The soil samples were collected in each quadrat, and the soil samples were collected in layers of 0–10 cm, 10–30 cm, and 30–50 cm with a soil sampler (with a diameter of 5 cm). The soil in the same layer was mixed into one soil sample. The collected soil samples were placed in sterile ziplock bags, quickly placed in sealed ice pack containers for cold storage, and then brought back to the laboratory for storage at 4 ℃. Then, two treatment samples were prepared. A part of the soil samples was used to determine soil organic carbon and its active carbon components. Soil organic carbon (SOC) was determined by the high-temperature external thermal potassium dichromate oxidation-capacity method. Soil-dissolved organic carbon (DOC) was determined by 0.5 mol·L^−1^K_2_SO_4_ extraction [30]. Easily oxidized organic carbon (EOC) was determined by the potassium permanganate oxidation-colorimetric method. Particulate organic carbon (POC) was determined by the sodium hexametaphosphate dispersion method [31]. Microbial biomass carbon (MBC) was determined by chloroform fumigation extraction [32]. The remaining samples were routinely processed and stored in sealed bottles after passing through a 2 mm sieve for analysis of other soil's physical and chemical properties. Soil pH was measured by a PHS-3 pH meter (ELTA32, Mettler-Toledo, Germany). At the same time, the ring knife soil was collected, and the fresh weight was weighed. After drying and weighing, the soil water content (SWC) and soil bulk density (SBD) were calculated. Total nitrogen (TN) and total phosphorus (TP) were determined by a German Vario EL III elemental analyzer.

### Data analysis

One-way ANOVA and multiple comparisons (α = 0.05) were performed for soil indexes in each soil layer of different plant communities. Duncan’s test was used as a statistical test. Dunnett’s T3 test was used for the statistical test when the data did not meet the homogeneity of variance. Pearson correlation analysis was conducted for soil physical and chemical properties and carbon component indexes. Soil physical and chemical properties include SWC, SBD, EC, pH, TN, TP (Table 1). The stepwise regression analysis method was used to establish the multiple regression equation of soil factors to each component's organic carbon content. The influence degree and relative contribution of each factor to soil organic carbon and its active carbon components were compared and analyzed. Data processing and analysis were carried out in Excel 2003 and SPSS 22.0, and plotting was completed in Origin 2019 software.

**Table 1:** Common parameters and their abbreviations

Parameter	Abbreviations	Units
*Suaeda corniculata*	*SC*	
*Salicornia europaea*	*SE*	
*Tamarix ramosissima*	*TR*	
*Phragmites australis*	*PA*	
Soil organic carbon	SOC	*g*/kg
Dissolved organic carbon	DOC	*g*/kg
Easily oxidized organic carbon	EOC	*g*/kg
Particulate organic carbon	POC	*g*/kg
Microbial biomass carbon	MBC	*g*/kg
soil water content	SWC	%
soil salinity	EC	%
Soil bulk density	SBD	*g*/cm^3^
Total nitrogen	TN	%
Total phosphorus	TP	%
Aboveground biomass	UB	*g* m^–2^
Average height	AH	cm
average density	D	branch*·*m^−2^
vegetation coverage	FVC	%

## Results

### Characteristics of soil physicochemical properties of different vegetation communities

The soil physicochemical indexes showed significant changes under different soil layers of four plant communities in salt marsh wetlands (Table 2). SWC in 0–50 cm soil from high to low was *PA*(39.54 ± 1.32%) > *SE*(33.71 ± 1.95%) > *SC*(23.26 ± 1.04%) > *TR* (21.66 ± 1.56%). The SWC of different soil layers under the same vegetation community was different and the SWC of each vegetation community showed an increasing trend with the increase of soil depth. The SBD of different soil layers in different vegetation communities was different, and ranged from 1.36 *g*/m^2^ to 2.15 *g*/m^2^. The SBD of 0–50 cm soil from high to low was *TR* (2.03 ± 0.05 *g*/m^2^) > *PA* (1.57 ± 0.02 *g*/m^2^) > *SC*(1.51 ± 0.02 *g*/m^2^) > *SE*(1.38 ± 0.03 *g*/m^2^)(Table 2). The SBD of each community decreased with the increase of soil depth. The soil salinity of 0–50 cm in *the SE* community was relatively high, with a value of 21.43 ± 0.31 *g*/kg, while that of the *TR* community was low, with a value of 6.72 ± 0.31 *g*/kg. EC in each community decreased with the increase in soil depth. Soil pH values ranged from 6.53 to 7.81, and the soil pH values of the *SE* community were significantly lower than those of the other three plant communities, and the differences among the other three plant communities were small. TN content in 0–50 cm soil from high to low was *SE* (0.38 ± 0.01) > *SC* (0.37 ± 0.01) > *PA(0.35 ± 0.01)* > *TR* (0.29 ± 0.01), and TN in *SC* and *SE* community was higher in 10–30 cm. TN in *PA* and *TR* was higher at 0–10 cm. Soil total phosphorus (TP) content in 0–50 cm soil from high to low was *SE* (0.56 ± 0.01) > *SC* (0.55 ± 0.01) > *PA* (0.54 ± 0.01) > *TR* (0.53 ± 0.01). There was little difference in soil TP among vegetation communities.

**Table 2:** Soil physical and chemical properties of the four vegetation communities in salt marsh wetland

Plant community	Soil layer	SWC%	SBD (*g*/m^2^)	EC%	ph	TN%	TP%
*SC*	0–10 cm	20.97 ± 0.96	1.54 ± 0.02	23.13 ± 0.72	7.73 ± 0.07	0.37 ± 0.01	0.58 ± 0.01
	10–30 cm	23.57 ± 1.02	1.51 ± 0.02	13.43 ± 0.67	7.69 ± 0.08	0.49 ± 0.01	0.53 ± 0.01
	30–50 cm	25.24 ± 1.16	1.49 ± 0.02	10.10 ± 0.56	7.60 ± 0.07	0.49 ± 0.01	0.53 ± 0.01
	0–50 cm	23.26 ± 1.04	1.51 ± 0.02	15.55 ± 0.48	7.67 ± 0.07	0.37 ± 0.01	0.55 ± 0.01
*SE*	0–10 cm	31.71 ± 1.94	1.43 ± 0.03	31.22 ± 0.66	6.77 ± 0.09	0.38 ± 0.01	0.58 ± 0.01
	10–30 cm	33.30 ± 2.13	1.36 ± 0.03	21.99 ± 0.33	6.64 ± 0.07	0.50 ± 0.01	0.53 ± 0.01
	30–50 cm	36.10 ± 1.89	1.36 ± 0.03	11.07 ± 0.35	6.53 ± 0.06	0.25 ± 0.01	0.55 ± 0.01
	0–50 cm	33.71 ± 1.95	1.38 ± 0.03	21.43 ± 0.31	6.64 ± 0.07	0.38 ± 0.01	0.56 ± 0.01
*TR*	0–10 cm	19.35 ± 1.52	2.15 ± 0.06	8.15 ± 0.42	7.81 ± 0.06	0.45 ± 0.01	0.56 ± 0.01
	10–30 cm	21.79 ± 1.59	2.08 ± 0.05	6.62 ± 0.29	7.72 ± 0.07	0.31 ± 0.01	0.53 ± 0.01
	30–50 cm	23.84 ± 1.61	1.87 ± 0.06	5.38 ± 0.34	7.64 ± 0.06	0.11 ± 0.01	0.52 ± 0.01
	0–50 cm	21.66 ± 1.56	2.03 ± 0.05	6.72 ± 0.31	7.73 ± 0.06	0.29 ± 0.01	0.53 ± 0.01
*PA*	0–10 cm	37.61 ± 1.43	1.61 ± 0.02	15.09 ± 1.00	7.65 ± 0.08	0.57 ± 0.01	0.57 ± 0.01
	10–30 cm	39.42 ± 1.31	1.57 ± 0.02	7.94 ± 0.24	7.63 ± 0.07	0.37 ± 0.01	0.53 ± 0.01
	30–50 cm	41.58 ± 1.24	1.53 ± 0.02	3.51 ± 0.12	7.57 ± 0.07	0.11 ± 0.01	0.50 ± 0.01
	0–50 cm	39.54 ± 1.32	1.57 ± 0.02	8.84 ± 0.42	7.62 ± 0.07	0.35 ± 0.01	0.54 ± 0.01

(mean ± SE). Different lowercase letters in the same column indicate significant differences among plots (*p* < 0.05). The full definitions of SC, SE, SWC and other parameter abbreviations are shown in Table 1

### Analysis of biological characteristics of different plant communities

There were significant differences in the biological characteristics of different vegetation communities in the Qinwangchuan salt marsh wetland (Table 3) (*P* < *0.05*). The FVC of each vegetation type ranged from 74.27% to 88.60%, and the plant average height (AH), coverage (FVC), and aboveground biomass (UB) of *TR* and *PA* communities were relatively high. The density (D) of the *TR* community was relatively low, with a value of 0.46 branch·m^−2^. The AH and UB of *SC* and *SE* communities were relatively low and the density was relatively high, which were 160.13 branch·m^−2^ and 105.27 branch·m^−2^, respectively.

**Table 3:** Biological characteristics of four typical vegetation communities in salt marsh wetland (mean ± SE)

phytocoenosium	AH (cm)	D (branch·m^−2^)	FVC (%)	UB (*g*/m^2^)
*SC*	25.23 ± 1.13	160.13 ± 6.41	74.27 ± 3.08	505.93 ± 33.37、
*SE*	19.08 ± 1.25	105.27 ± 8.22	84.53 ± 1.87	856.33 ± 57.76
*TR*	276.93 ± 9.23	0.46 ± 0.04	86.13 ± 2.40	1470.40 ± 66.15
*PA*	193.44 ± 6.29	287.20 ± 9.26	88.60 ± 1.50	2216.67 ± 81.31

Different lowercase letters in the same column indicate significant differences among plots (*p* < 0.05). For a definition of the parameter abbreviations see Table 1

### Characteristics of soil organic carbon and active carbon components under different vegetation communities

#### Characteristics of soil organic carbon under different vegetation communities

**Fig. 2:**
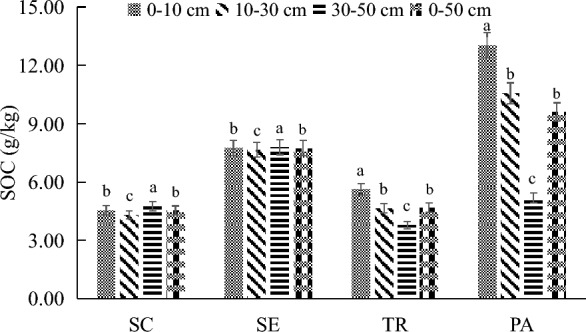
Characteristics of soil organic carbon content in different vegetation communities in Qinwangchuan salt marsh wetland. Different lowercase letters in the same column indicate significant differences among plots (*p* < 0.05). *SC* Suaeda corniculata, *SE*, Salicornia europaea, *TR* Tamarix ramosissima, *PA* Phragmites australis

Figure 2 shows that there are significant differences in soil organic carbon among the four vegetation communities in the salt marsh wetland (*P* < 0.05). From high to low soil organic carbon (SOC) in 0–50 cm soil was *PA* (9.60 ± 0.32 *g*/kg) > *SE* (7.75 ± 0.18*g*/kg) > *TR* (4.96 ± 0.18 *g*/kg) > *SC* (4.55 ± 0.11 *g*/kg). The SOC values of the *PA* community in 0–10 cm, 10–30 cm, and 30–50 cm soil layers were 13.04 ± 0.45 *g*/kg, 10.58 ± 0.36 *g*/kg and 5.19 ± 0.24 *g*/kg, respectively. The SOC of the *TR* community was 0–10 cm (5.63 ± 0.26 *g*/kg) > 10–30 cm (4.65 ± 0.18 *g*/kg) > 30–50 cm (3.79 ± 0.18 *g*/kg) in vertical profile. In the vertical profile, the soil organic carbon content of the *PA* and *TR* communities decreased with the increase in soil depth. The SOC values of the *SE* community was 30–50 cm (7.81 ± 0.20 *g*/kg) > 0–10 cm (7.77 ± 0.20 *g*/kg) > 10–30 cm (7.66 ± 0.17 *g*/kg) in descending order. The SOC values of the *SC* community were lower in 0–10 cm, 10–30 cm, and 30–50 cm soil layers, which were 4.57 ± 0.13 *g*/kg, 4.32 ± 0.12 *g*/kg, and 4.76 ± 0.10 *g*/kg, respectively. In the vertical profile, the SOC of the *SE* and *SC* community decreased first and then increased with the increase of soil depth.

#### Characteristics of soil active organic carbon components under different vegetation communities

**Fig. 3:**
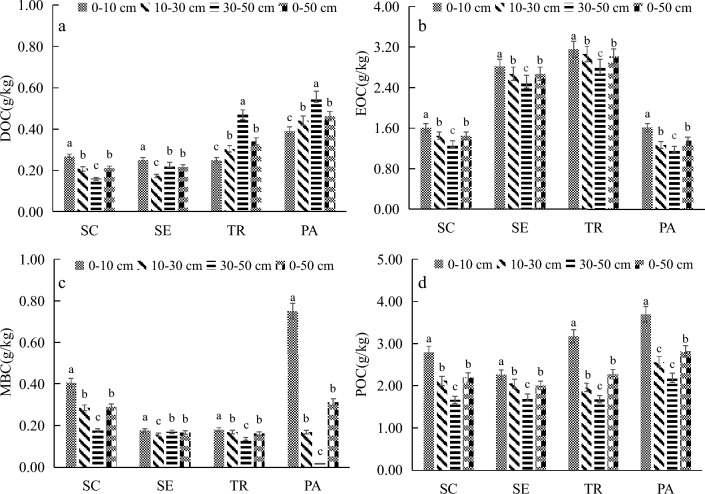
Active carbon components of soil organic carbon under different vegetation in Qinwangchuan salt marsh wetland (a, DOC. b, EOC.c, MBC. d, POC). Different lowercase letters in the same column indicate significant differences among plots (*p* < 0.05). *SC* Suaeda corniculata, *SE* Salicornia europaea, *TR* Tamarix ramosissima, *PA* Phragmites australis. For definition of the parameter abbreviations see Table 1

Soil dissolved organic carbon (DOC), easily oxidized organic carbon (EOC), particulate organic carbon (POC), and soil microbial biomass carbon (MBC) were significantly different among the four plantation communities (Fig. 3). Figure 3a shows that the DOC in 0–50 cm vegetation community was as follows *PA* (0.46 ± 0.01 *g*/kg) > *TR* (0.34 ± 0.01 *g*/kg) > *SE* (0.22 ± 0.01 *g*/kg) > *SC* (0.21 ± 0.01 *g*/kg). In the vertical section, DOC of the *SC* community was 0–10 cm (0.27 ± 0.02 *g*/kg) > 10–30 cm (0.21 ± 0.01 *g*/kg) > 30–50 cm (0.16 ± 0.01 *g*/kg). The DOC values of the *SE* community in 0–10 cm, 10–30 cm and 30–50 cm soil layers were 0.25 ± 0.02 *g*/kg, 0.17 ± 0.01 *g*/kg and 0.23 ± 0.01 *g*/kg, respectively. The DOC of the *PA* and *TR* communities was lower in the 0–10 cm soil layer, and its value was 0.39 ± 0.01 *g*/kg and 0.25 ± 0.02 *g*/kg, respectively. The DOC of the *PA* and *TR* communities was higher in the 30–50 cm soil layer. Their values were 0.56 ± 0.01 *g*/kg and 0.47 ± 0.01 *g*/kg, respectively. In the vertical profile, the DOC of the *PA* and *TR* community increased with the increase in soil depth (Fig. 3a). The EOC in 0–50 cm vegetation community showed *TR* (3.01 ± 0.05 *g*/kg) > *SE* (2.67 ± 0.07 *g*/kg) > *SC* (1.45 ± 0.07 *g*/kg) > *PA* (1.36 ± 0.04 *g*/kg) (Fig. 3b).

The MBC in 0–50 cm vegetation community showed *PA* (0.31 ± 0.01 *g*/kg) > *SC* (0.29 ± 0.01 *g*/kg) > *SE* (0.17 ± 0.01 *g*/kg) > *TR* (0.16 ± 0.01 *g*/kg) (Fig. 3c). The MBC of the *SE* community was different in 0–10 cm, 10–30 cm, and 30–50 cm soil layers, and the values were 0.18 ± 0.01 *g*/kg, 0.16 ± 0.01 *g*/kg and 0.17 ± 0.01 *g*/kg, respectively. The MBC of the *SC* community was 0–10 cm (0.41 ± 0.01g/kg) > 10–30 cm (0.29 ± 0.02 g/kg) > 30–50 cm (0.18 ± 0.01 g/kg). The microbial biomass carbon (MBC) of the *PA* community was significantly higher in the 0–10 cm surface layer than in the 10–30 cm and 30–50 cm soil layers, and decreased with the increase of soil depth. The MBC of the *TR* community was different in 0–10 cm, 10–30 cm, and 30–50 cm soil layers, and the values were 0.18 ± 0.01 *g*/kg, 0.17 ± 0.01 *g*/kg, and 0.14 ± 0.01 *g*/kg, respectively (Fig. 3c). The particulate organic carbon (POC) in 0–50 cm vegetation community was showed *PA* (2.81 ± 0.06 *g*/kg) > *TR* (2.27 ± 0.06 *g*/kg) > *SC* (2.19 ± 0.08 *g*/kg) > *SE* (2.01 ± 0.07 *g*/kg). In the vertical profile, EOC and POC decreased with the increase of soil depth (Fig. 3d).

### Analysis of influencing factors of soil organic carbon and active carbon components

#### Correlation analysis of soil organic carbon and active carbon components with vegetation community and soil factors

**Fig. 4:**
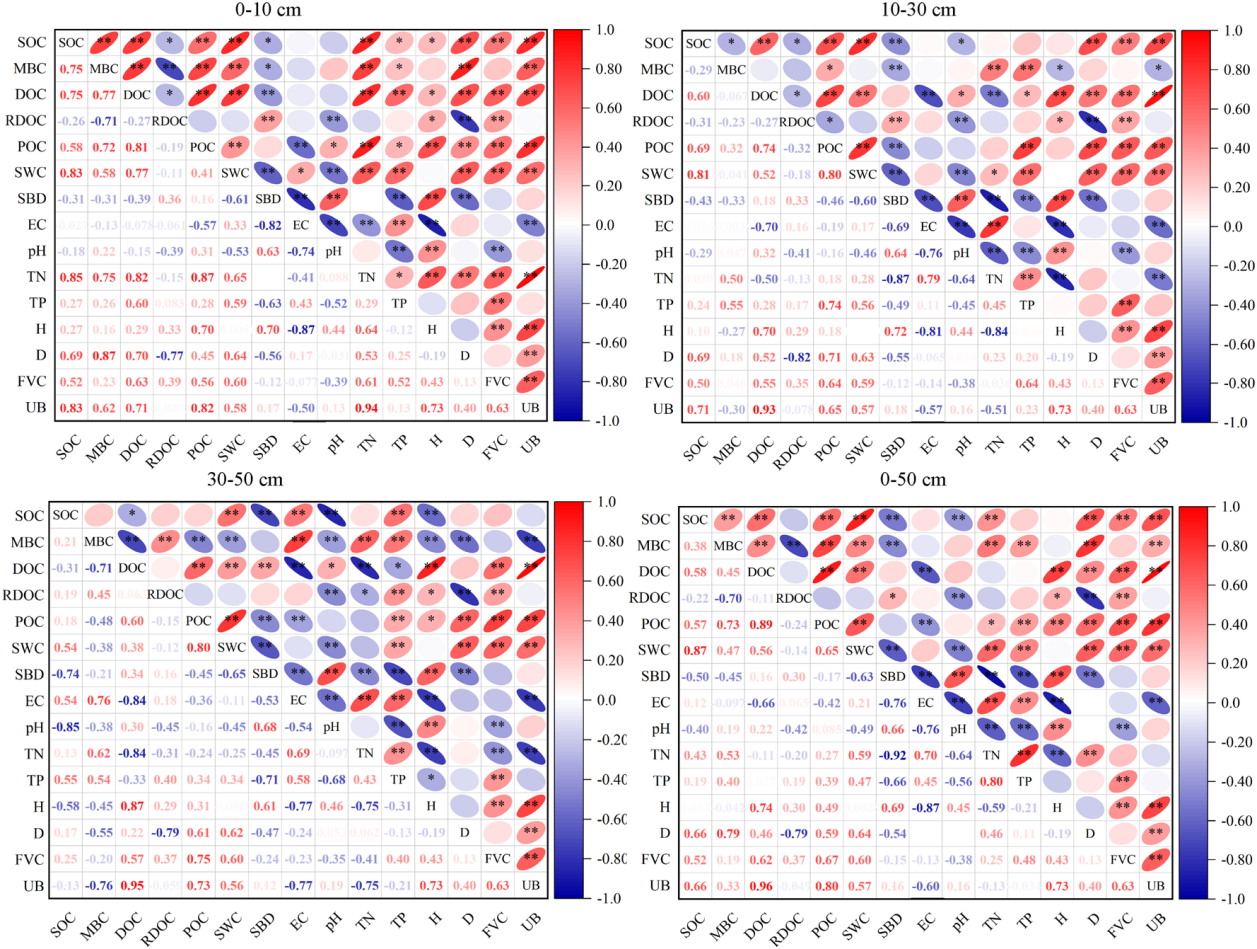
Correlation analysis of soil organic carbon and active carbon components with vegetation community and soil factors. *p < 0.05 (bilateral); **p < 0.01 (bilateral). For a definition of the parameter abbreviations see Table 1

Figure 4 shows the effects of soil physical and chemical properties and vegetation community characteristics on SOC and active carbon components by Pearson correlation analysis. The influencing factors of soil organic carbon and carbon components are quite different, and the influencing factors of the same carbon component are also different in different soil layers (Fig. 4). SOC was positively correlated with DOC, POC, and MBC. SOC greatly affected the soil's active organic carbon content, and SOC was negatively correlated with EOC. SOC in 0–50 cm soil layer was positively correlated with SWC, TN, TP, D, FVC, and UB (*P* < *0.01*), and the correlation decreased with the increase in soil depth. There was a significant negative correlation between SOC and SBD (*P* < *0.01*), and the correlation between SOC and SBD increased with the increase in soil depth. SOC was not significantly correlated with pH and plant height in 0–10 cm and 10–30 cm soil layers, but was significantly negatively correlated with pH and plant height in 30–50 cm soil layers (*P* < *0.01*). Soil factors significantly related to active carbon components were more in 0–10 cm and 10–30 cm, and the significantly related factors gradually decreased with the increase of soil depth. DOC was positively correlated with SWC, TN, D, FVC and UB (*P* < *0.01*), and the correlation decreased with the increase of soil depth. There was a significant negative correlation between DOC and EC *(P* < *0.01*), and the correlation was enhanced with the increase in soil depth. EOC was positively correlated with SBD and FVC (*P* < *0.05*), and the correlation decreased with the increase in soil depth. POC was positively correlated with TP, AD, FVC and UB, and negatively correlated with EC in 0–10 cm and 10–30 cm soil. The environmental factors significantly related to POC and EOC decreased with the increase in soil depth.

#### Decomposition effects of vegetation and soil factors on soil organic carbon and active carbon components

Due to the strong correlation between soil factors, collinearity may occur during regression analysis, and the variation range of the quantity of each soil factor is different. Therefore, stepwise regression analysis cannot directly reflect the contribution of each environmental factor to the content of soil organic carbon and active carbon components. The path coefficient was calculated by the method of the standardized regression coefficient. The correlation coefficient was decomposed into direct path coefficient and indirect path coefficient, which could directly reflect the influence of various environmental factors on soil organic carbon and active carbon components. The statistical regression model of soil organic carbon, active carbon components and other soil factors was obtained by stepwise regression analysis (1) – (5):

1 $${\text{SOC}} = {24}.{51} + 0.00{\text{8SWC}} - {2}.{\text{319SBD}} + 0.{\text{126EC}} - 0.{\text{747pH}} + {1}0.{\text{786TN}} - {32}.{\text{64TP}} + 0.00{\text{1D}} - 0.00{\text{5FVC}} + 0.00{\text{3UB}}$$

2 $${\text{MBC}} = - {1}.{141} - 0.00{\text{1SWC}} - 0.00{\text{3SBD}} + 0.0{\text{84pH}} + {1}.{1}0{\text{8TN}} + 0.{\text{549TP}}$$

3 $${\text{DOC}} = - 0.0{28} + 0.00{\text{2SWC}} + 0.0{\text{32SBD}} - 0.0{\text{17 pH}} - 0.0{\text{5 TN}} + 0.{\text{455 TP}}$$

4 $${\text{EOC}} = {3}.{721} + 0.00{\text{4SWC}} + 0.{\text{983SBD}} + 0.0{\text{18EC}} - 0.{\text{835pH}} - {3}.{\text{346TN}} + {6}.{\text{337TP}} - 0.00{\text{4D}} + 0.00{\text{8 FVC}}$$

5 $${\text{POC}} = {3}.{721} + 0.00{\text{4SWC}} + 0.{\text{983SBD}} - 0.{\text{118PH}} - {3}.{\text{346TN}} + {6}.{\text{337TP}} - 0.00{\text{4D}} + 0.00{\text{8FVC}}$$

The correlation between vegetation community characteristics, soil factors, and SOC content was in the order of SWC > UB > D > FVC > SBD > TN > pH > TP > EC (Table 4). The direct path coefficients of UB and TP are both larger than their connected path coefficients, indicating that the main contribution of UB and TP to SOC content is the direct effect. UB has the largest direct effect on SOC and shows a positive effect, while TP has the largest direct negative effect on SOC. The indirect path coefficient of SOC, SWC, SBD, EC, PH, TN, FVC, and other factors is greater than its direct path coefficient. The results showed that SWC, SBD, EC, PH, TN and other environmental factors contributed to SOC content mainly through the indirect effect of affecting other factors. SWC, SBD, EC, and PH mainly have indirect negative effects through UB and indirect positive effects through TP. TN has an indirect positive effect through UB and a negative effect through TP.

**Table 4:** Decomposition of the simple correlation coefficient between soil organic carbon and vegetation-soil factors in a salt marsh wetland

Items	Index	correlation coefficient	Direct path factor	Indirect path factor										
				SWC	SBD	EC	PH	TN	TP	H	D	FVC	UB	合计
SOC	SWC	0.868	0.033		0.177	0.068	0.082	− 0.110	0.197	0.028	− 0.014	0.011	− 0.480	− 0.041
	SBD	-0.501	-0.282	− 0.021		− 0.251	0.128	− 0.170	0.304	0.043	− 0.022	0.017	− 0.742	− 0.714
	EC	0.121	0.331	0.007	− 0.058		0.127	− 0.170	0.304	0.043	− 0.022	0.017	− 0.740	− 0.492
	PH	-0.395	− 0.168	− 0.016	0.138	− 0.163		− 0.143	0.255	0.036	− 0.018	0.015	− 0.623	− 0.518
	TN	0.434	0.224	0.019	− 0.166	0.195	− 0.099		− 0.321	− 0.046	0.023	− 0.018	0.783	0.371
	TP	0.195	− 0.401	0.016	− 0.133	0.157	− 0.079	0.106		0.012	− 0.006	0.005	− 0.201	− 0.126
	D	0.661	0.029	0.021	− 0.179	0.210	− 0.107	0.142	− 0.255	− 0.036		− 0.003	0.127	− 0.079
	FVC	0.519	− 0.023	0.020	− 0.169	0.198	− 0.101	0.134	− 0.240	− 0.034	0.017		0.615	0.441
	UB	0.665	0.978	0.019	− 0.162	0.190	− 0.096	0.128	− 0.230	− 0.033	0.017	− 0.013		− 0.180

For a definition of the parameter abbreviations see Table 1

It can be seen from Table 5 that the correlation between soil factors and MBC content from large to small was TN > SWC > SBD > TP > pH. The direct path coefficients of TN and pH are both larger than the connected path coefficients, indicating that the main contribution of TN and pH to MBC content is the direct effect, and the effect is positive. The indirect path coefficients of SWC, SBD, and TP were larger than those of their direct path coefficients, indicating that the main contribution of SWC, SBD, and TP to MBC content was an indirect effect through influencing other factors. SWC and SBD have indirect negative effects mainly through pH, TN, and D. TN has an indirect positive effect through D.

**Table 5:** Decomposition of the simple correlation coefficient between soil active organic carbon components and vegetation-soil factors in saltmarsh wetland

Items	Index	correlation coefficient	Direct path factor	Indirect path factor										
				SWC	SBD	EC	PH	TN	TP	H	D	FVC	UB	合计
MBC	SWC	0.468	− 0.080		0.006	0.062	− 0.342	− 0.418	− 0.122	− 0.149	− 0.372	− 0.044	0.080	− 1.299
	SBD	− 0.454	− 0.010	0.050		0.075	− 0.414	− 0.505	− 0.148	− 0.180	− 0.451	− 0.053	0.096	− 1.530
	PH	0.190	0.546	0.039	0.005	0.049		− 0.424	− 0.124	− 0.151	− 0.378	− 0.045	0.081	− 0.949
	TN	0.532	0.666	− 0.047	− 0.006	− 0.058	0.322		0.156	0.190	0.476	0.056	− 0.102	0.987
	TP	0.398	0.195	− 0.038	− 0.005	− 0.047	0.258	0.315		− 0.049	− 0.122	− 0.014	0.026	0.324
DOC	SWC	0.555	0.002		− 0.020	0.004	0.011	0.031	− 0.285			− 0.001		− 0.260
	SBD	0.155	0.032	− 0.001		0.005	0.013	0.038	− 0.345			− 0.001		− 0.292
	EC	− 0.658	− 0.006	0.000	0.007		0.013	0.038	− 0.344			− 0.001		− 0.288
	PH	0.223	− 0.017	− 0.001	− 0.016	0.003		0.032	− 0.290			− 0.001		− 0.272
	TN	− 0.107	− 0.050	0.001	0.019	− 0.004	− 0.010		0.364			0.001		0.372
	TP	0.027	0.455	0.001	0.015	− 0.003	− 0.008	− 0.024						− 0.019
EOC	SWC	− 0.142	0.045		− 0.224	− 0.091	0.353	0.130	− 0.146	− 0.103	0.354	− 0.069	− 0.039	0.166
	SBD	0.295	0.357	− 0.028		− 0.110	0.427	0.158	− 0.177	− 0.124	0.428	− 0.083	− 0.047	0.443
	EC	0.065	0.145	0.009	0.074		0.426	0.157	− 0.176	− 0.124	0.427	− 0.083	− 0.047	0.663
	PH	− 0.424	− 0.563	− 0.022	− 0.175	− 0.071		0.132	− 0.148	− 0.104	0.359	− 0.070	− 0.039	− 0.139
	TN	− 0.199	− 0.208	0.027	0.211	0.086	− 0.332		0.187	0.131	− 0.452	0.088	0.050	− 0.005
	TP	0.189	0.233	0.021	0.169	0.069	− 0.266	− 0.098		− 0.034	0.116	− 0.023	− 0.013	− 0.059
	D	− 0.795	-0.564	0.029	0.227	0.092	− 0.358	− 0.132	0.148	0.104		0.014	0.008	0.132
	FVC	0.370	0.110	0.027	0.214	0.087	− 0.337	− 0.125	0.140	0.098	− 0.338		0.039	− 0.195
POC	SWC	− 0.142	0.045		− 0.224	− 0.091	0.353	0.130	− 0.146	− 0.103	0.354	− 0.069	− 0.039	0.166
	SBD	0.295	0.357	− 0.028		− 0.110	0.427	0.158	− 0.177	− 0.124	0.428	− 0.083	− 0.047	0.443
	PH	− 0.424	− 0.563	− 0.022	− 0.175	− 0.071		0.132	− 0.148	− 0.104	0.359	− 0.070	− 0.039	− 0.139
	TN	− 0.199	− 0.208	0.027	0.211	0.086	− 0.332		0.187	0.131	− 0.452	0.088	0.050	− 0.005
	TP	0.189	0.233	0.021	0.169	0.069	− 0.266	− 0.098		− 0.034	0.116	− 0.023	− 0.013	− 0.059
	D	– 0.795	− 0.564	0.029	0.227	0.092	− 0.358	− 0.132	0.148	0.104		0.014	0.008	0.132
	FVC	0.370	0.110	0.027	0.214	0.087	− 0.337	− 0.125	0.140	0.098	− 0.338		0.039	− 0.195

For a definition of the parameter abbreviations see Table 1

The correlation between soil factors and DOC content was EC > SWC > pH > SBD > TN > TP (Table 5). The direct path coefficient of TP was greater than the connected path coefficient, indicating that the main contribution of TP to DOC content was a direct effect and a positive effect. The indirect path coefficients of EC, SWC, pH, SBD, and TN were larger than those of their direct path coefficients, indicating that the main contribution of EC, SWC, pH, SBD, and TN to DOC content was indirect effect by influencing other factors. Among them, EC, SWC, pH and SBD mainly produce indirect negative effects through TP, while TN produces indirect positive effects through TP (Table 5).

The correlations of vegetation community characteristics, soil physicochemical factors with EOC content and POC content were as follows D > pH > FVC > SBD > TN > TP > SWC > EC (Table 5). The direct path coefficients of PH and D on EOC and POC were larger than the connected path coefficients, indicating that the main contribution of PH and D to EOC and POC content was the direct effect, and both showed a negative effect. The indirect path coefficients of SWC, SBD, EC, TN, TP, and FVC were larger than their direct path coefficients, indicating that the main contribution of these factors to EOC content was indirect effect through influencing other factors. SWC, SBD and EC have indirect positive effects by influencing PH and D, while TN, TP and FVC have indirect negative effects by influencing PH. In general, the interaction between vegetation community characteristics and soil physical and chemical factors jointly affected the content of soil organic carbon and its active components.

## Discussion

### Different salt marsh wetland vegetation types of soil organic carbon and active carbon components characteristics

Soil organic carbon mainly comes from the return of plant litter elements and the metabolism of root exudates [33]. The quality and quantity of soil organic carbon input are different due to the difference in aboveground biomass, litter, and exudate. It directly or indirectly determines the decomposition rate and decomposition mode of soil organic carbon, and eventually leads to the change of soil organic carbon and active carbon components [34]. This study found that soil organic carbon content showed the order of *PA* > *SE* > *TR* > *SC* community (Fig. 2). Plant community characteristics (FVC, D, and UB) were significantly positively correlated with SOC (*P* < *0.05*), and UB had the largest direct positive effect on SOC (Fig. 4), which was consistent with Nie [35]. Wetland plant *Phragmites australis* is the dominant species in the study area. *PA* has the characteristics of high carbon input and low carbon output, high productivity and salinity tolerance. The wetland is in a wet water-saturated state, which inhibits the decomposition of organic matter and has a large effect on soil carbon sequestration. In addition, the FVC, D, and UB of the *PA* community were significantly higher than those of other vegetation communities (Table 3). This provides more litter to the soil and increases the content of organic carbon and active carbon components in the soil, which is consistent with the results of Gou [36]. The growth and development of plant communities play an important role in soil organic carbon. The relative deposition of soil under the flourishing growth of vegetation and the return of vegetation litter into the soil is conducive to the preservation of soil nutrients and the accumulation of wetland soil organic carbon. Soil organic carbon in *TR* and *PA* communities was concentrated in the soil surface layer (0–10 cm), and soil organic carbon content decreased with the increase of soil depth (Fig. 2). This is because the 0–10 cm depth soil layer has a large accumulation of plant litter, a high input of organic matter decomposition, and strong microbial activity. With the increase in soil depth, the input of litter decreased, the microbial activities decreased sharply, and the organic carbon content began to decrease significantly. The significant positive correlations between plant community characteristics (FVC, D, and UB) and soil organic carbon (*P* < *0.05*) decreased with the increase in soil depth (Fig. 4), which was consistent with the results of Mukhopadhyay [37]. SOC in *SE* and *SC* communities showed an inverted V-shaped distribution, with the highest content in the 10–30 cm soil layer (Fig. 2). Because the roots of *SC* and *SE* are mainly concentrated in the soil of 10–30 cm, the roots intertwine with each other and connect root particles to release secretions, which leads to changes in the nature of rhizosphere soil and affects soil aggregation. In addition, a large number of dead roots provide rich carbon sources for the soil. This is similar to the research results of Zi [38], the denser the plant roots are distributed, the higher the content of organic carbon in the soil of 10–30 cm soil layer.

Soil active organic carbon is an important part of soil organic carbon, which can reflect the sensitivity of the soil carbon pool. The plant community injects photosynthally fixed organic carbon into soil through litter, turnover of fine roots, and root exudates [39, 40]. The quantity and quality of litter transported to the soil by different vegetation types and the root exudates were different, resulting in significant differences in soil active carbon components (MBC, EOC, DOC, and POC) in different soil layers of different plant communities [41]. The contents of soil active carbon components MBC, DOC and POC in the *PA* community were higher (Fig. 3). This is mainly because the *PA* community is located in the seasonally flooded waterlogged swamp area, where vegetation is flourishing and more litter provides a large amount of carbon source for soil microorganisms. Moreover, the soil permeability is relatively good, which promotes the propagation of microorganisms and can significantly increase MBC. DOC is a source of organic carbon that can be used directly by soil microbes. It moves quickly through the soil and is easily broken down and mineralized, so it is easily lost [42]. Flooding can improve the dissolution of soil organic carbon and lead to the dispersion of aggregates, thus increasing the amount of dissolved organic carbon. DOC increases with increasing soil depth, possibly because DOC includes different orders of organic matter suspended and deposited in soil solutions. On the one hand, many large organic combinations of soil particles and plant litter are enriched in the soil surface, and their decomposition ability is relatively weak, and the accumulation and conversion rate of DOC in the soil is low. On the other hand, the leaching effect of soil and the evaporation effect of deep soil was weak, which enhanced the adsorption and retention of DOC in deep soil, this is consistent with the conclusions of Liu [43]. The higher the particulate organic carbon, the higher the unstable part of soil organic carbon. Due to differences in plant communities, root distribution, water status, pH value, and SOC, the distribution of MBC, DOC and POC in soil layers was also different, which was consistent with the results of Guan and Sainepo [10, 44]. The SWC of the *TR* community is low, soil litter input is relatively less, SOC is easy to oxidized, and microbial growth and reproduction are inhibited, with relatively few types and quantities. The contents of MBC, DOC and POC in the *TR* community were lower, but the EOC was significantly higher than that of other communities. This is because the spatial structure of the TR community (large crown width, high plant height, and deep litter leaf layer) not only plays a shading role on the soil under the crown, reduces the evaporation of soil water, but also reduces the loss of soil EOC caused by leaching and precipitation scouring. Leaching and leaching of topsoil erosion are the decisive factors affecting the difference of EOC. Since soil active organic carbon mainly comes from plant litter, soil humus, microorganisms, roots and their exudates, exudates produced by roots and root exudates will increase SOC content. At the same time, more distribution of surface roots and litter can provide more carbon sources for microorganisms, which is conducive to microbial growth and reproduction. With the deepening of the soil layer, soil organic carbon content and underground biomass decreased, resulting in a significant decrease in soil active organic carbon. The contents of different active organic carbon components reflected that different plant communities had a great influence on the organic carbon content of salt marsh wetlands.

### Effects of soil physical and chemical properties on soil organic carbon and active carbon components

The correlation analysis showed that the correlation between soil organic carbon and the active carbon component of salt marsh wetland reached a significant level (*P* < *0.01*), indicating that soil active organic carbon was largely dependent on SOC. SOC was significantly positively correlated with the contents of MBC, DOC, and POC (P < 0.01), and negatively correlated with EOC. The correlation intensity between soil organic carbon and active carbon components in different soil layers was different, which was consistent with the conclusion of Xu [45]. The difference in soil physical and chemical properties can also lead to a change of the soil organic carbon production by affecting microbial activity and the soil organic carbon mineralization process. It was found that SOC, DOC, POC, and MBC were positively correlated with SWC and TN, and negatively correlated with SBD. This is mainly because of the increase of soil moisture for plant litter decomposition and soil microbial activity, which would be helpful to the accumulation of soil organic carbon components together [46]. The higher the soil moisture content, and soil aeration, the less number of aerobic bacteria, SOC mineralization process is abated, the organic material input is greater than the output, the long-term accumulation of soil organic carbon content increased [29, 47]. On the other hand, water also has indirect effects on soil organic carbon and active carbon components by affecting plant distribution and biomass size. The influence of nitrogen on soil organic carbon is mainly achieved by controlling the rate of organic carbon mineralization and plant growth. It is found that TN is significantly positively correlated with SOC and POC, and has a strong direct positive effect on MBC content. This is mainly because the increase of total nitrogen will promote plant growth and lead to the increase of nitrogen content in plant leaves, which will reduce the C/N ratio of litter, thus promoting the decomposition of litter, and eventually affecting the content of organic carbon in the soil. Secondly, TN can promote a significant increase in plant leaf length and leaf width in the short term, and then increase the quantity and quality of litter, increasing organic matter input. TN is positively correlated with organic carbon and active carbon components, which is consistent with the conclusion reached by Lu [48]. SBD is an important index reflecting soil's physical state. Higher SBD leads to lower soil porosity, resulting in worse soil permeability and aeration, which is not conducive to the survival of microorganisms and the growth of plant roots, and reduces the input of litter, thus affecting the content of organic carbon in the soil. SBD was significantly negatively correlated with SOC and MBC, which was consistent with the conclusions of Yan [49]. PH has a strong direct effect on SOC and active carbon components, and pH is significantly negatively correlated with SOC, MBC and EOC, which is consistent with the existing research conclusions of Huang [50] (Tables 4 and 5). PH directly affects the type, quantity and activity of soil microorganisms, thus affecting the soil carbon conversion process. Microorganisms are only suitable for activities in a neutral environment. Too high or too low PH will have adverse effects on the growth and reproduction of microorganisms, affect the normal growth of plants, reduce the input of litter, and then affect the content of soil organic carbon and active carbon components. PH directly affects the type, quantity and activity of soil microorganisms, thus affecting soil carbon conversion process. The difference in TP between different vegetation types is relatively small, which may be related to the poor migration of phosphorus itself and the easy existence of phosphorus in the soil as a deposition form. Soil organic carbon and active organic carbon content were positively correlated with TP content, but the correlation was not significant, which was similar to Jin’s research results [51]. The indirect effects of SWC, SBD, and EC on the contents of SOC, MBC, DOC, EOC, and POC by influencing other factors are similar to the research results of other scholars in Spohn [52] (Table 5). The difference in vegetation community characteristics and the interaction between soil physicochemical factors are the important factors driving the variation of soil organic carbon and active carbon components in different plant communities in salt marsh wetlands.

## Conclusions

There were significant differences in soil organic carbon and active organic carbon among different plant communities in the Qinwangchuan salt marsh wetland. Soil organic carbon content was PA (9.60 *g*/kg) > SE (7.75 *g*/kg) > CL (4.96 *g*/kg) > SC (4.55 *g*/kg), and DOC, POC, and MBC in the PA community was significantly higher than those in SE, CL and SC communities. In the vertical profile, SOC, DOC, POC, and MBC contents in the 0–10 cm soil layer were significantly higher than those in the 10–30 cm and 30–50 cm soil layers. There was a significant positive correlation between SOC and DOC, POC and MBC, and a negative correlation between SOC and EOC. SOC was positively correlated with vegetation community characteristics (height, coverage, density, and biomass) and soil environment (SWC, TN, TP, D, FVC, UB), but was negatively correlated with SBD as soil depth increased. There were differences in the correlation between soil active carbon components, vegetation community characteristics, and soil physicochemical properties in different soil layers. The results showed that the factors affecting the change of soil organic carbon and the stability of the carbon pool were complicated. In soil organic carbon protection, more attention should be paid to the improvement of soil properties and nutrients, and the improvement of plant community status also plays a positive role in the accumulation of soil organic carbon. There are two different ways that vegetation community characteristics and soil physicochemical properties can affect soil organic carbon and its components. First, TN, TP, pH, and UB have direct effects on SOC, DOC, EOC, and POC. Second, SWC, SBD, EC, and FVC have indirect effects on SOC, DOC, EOC, and POC contents by influencing other factors, indicating that in the process of soil organic carbon change, there are not only direct effects of each influencing factor but also indirect effects among influencing factors. In summary, soil organic carbon of different wetland plant communities in inland salt marsh wetlands has significant differences, but its internal driving mechanism is more complex. Therefore, in future studies, it is necessary to consider the influence of soil microorganisms, hydrological environment, and other factors more comprehensively to reflect the change process and mechanism of wetland soil carbon pool more deeply.

## Data Availability

All study-related data has been provided in the supporting information.
